# Heavy burden of soil-transmitted helminth infections in a remote and impoverished indigenous community of Honduras revealed by real time polymerase chain reaction

**DOI:** 10.1186/s40249-026-01454-5

**Published:** 2026-05-25

**Authors:** Maria Esther Araujo, Ana Sanchez, Gabriela Matamoros

**Affiliations:** 1 Microbiology Research Institute, Faculty of Science, National Autonomous University of Honduras, Tegucigalpa, Honduras; 2 Department of Health Sciences, Faculty of Applied Health Sciences, Brock University, 1812 Sir Isaac Brock Way, St. Catharines, ON L2S 3A1 Canada

**Keywords:** Soil-transmitted helminths, qPCR, Honduras, Hyperendemic, Prevalence

## Abstract

**Background:**

Soil-transmitted helminths (STH) remain highly prevalent in Honduras, disproportionately affecting rural and underserved populations. Diagnostic limitations, particularly for *Necator americanus* and *Strongyloides stercoralis*, hinder accurate burden estimation. This study aimed to determine the prevalence and species distribution of STH infections using real-time polymerase chain reaction (PCR) in a remote, resource-limited setting.

**Methods:**

A rapid, non-representative cross-sectional survey was conducted among children and adults in a remote community in the Honduran Moskitia. Stool samples were analyzed using multiplex real-time PCR. Prevalence was estimated with 95% confidence intervals. Differences between groups were assessed using Fisher’s exact test, and logistic regression models were used to explore associations of infection with age and sex. Ct values were summarized using medians and interquartile ranges.

**Results:**

Overall community STH prevalence was 85.2% [95% confidence intervals (*CI):* 78.6–92.3], with 72.2% (95% *CI:* 58.4–83.5) in adults and 98.1% (95% *CI:* 90.1–100) in school-aged children, who were predominantly infected with *Trichuris trichiura* (77.8%, 95% *CI:* 69.9–85.7), followed by *Ascaris lumbricoides* (50.0%, 95% *CI:* 40.6–59.4), *N. americanus* (29.6%, 95% *CI:* 21.0–38.2), and *S. stercoralis* (3.7%, 95% *CI:* 0.1–7.3). Younger age and female sex were identified as significant risk factors for *T. trichiura*.

**Conclusions:**

The extreme hyperendemicity of STH infections in the study community poses a serious health risk to its population, particularly children. These findings support the need for larger, systematically designed parasitological surveys that integrate sensitive diagnostic molecular tools to help precisely map STH epidemiology and guide control efforts in this and other underserved regions.

**Graphical Abstract:**

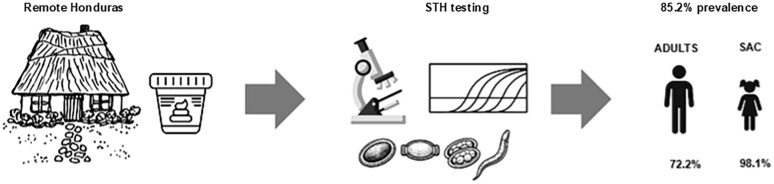

**Supplementary Information:**

The online version contains supplementary material available at 10.1186/s40249-026-01454-5.

## Background

Honduras is a small country located in Central America where helminthic infections-particularly those caused by soil-transmitted helminths (STH)-remain highly prevalent [1–3]. Four STH species are endemic to Honduras: the roundworm *Ascaris lumbricoides*, the whipworm *Trichuris trichiura*, the hookworm *Necator americanus*, and the threadworm *Strongyloides stercoralis.* As in other similar settings around the world, these intestinal parasites unequally affect rural and underserved communities [4–7], and disproportionately impact children, thus contributing to broader health issues such as chronic malnutrition, immune imbalance, and cognitive deficits [5, 8, 9]. At a larger societal scale, STH infections exert a heavy economic burden in affected communities [10].

Numerous Honduras-based studies have identified STH prevalence rates across different regions; the majority providing strong evidence of the continued transmission and distribution patterns of both *A. lumbricoides* and *T. trichiura* [2, 11, 12]. However, evidence for the other two species remains limited, as most investigations make use of the modified Kato-Katz (KK), a microscopy-based diagnostic method that is less reliable for detecting *Necator americanus*—owing to the fragility of its diagnostic eggs—[13], and entirely ineffective for *Strongyloides stercoralis* [14]. This diagnostic gap presents a significant challenge for accurately assessing the true burden of these species.

It is well-established that STH infections not only exhibit a strong preference for younger age groups but also display a distinct geographic pattern, disproportionately affecting populations based on environmental and infrastructural disparities [6]. In fact, studies show that STH national distribution in Honduras is influenced not only by young age and lower human development index [2], but also by geography [15–18]. Even among the Honduran poor, deeper levels of poverty have been observed as a function of geographical isolation, where topographical barriers and poor road infrastructure are directly linked to worse health outcomes and higher STH prevalence [11, 15].

Despite efforts to include remote and traditionally marginalized communities in STH research, Honduras lacks a systematic, territory-wide mapping of STH burden. One notable example is the Department of Gracias a Dios, home to the Honduran Moskitia, a region where biomedical research is difficult due to its extreme geographic isolation and safety concerns [19]. This persistent gap limits our understanding of STH distribution, potentially leaving high-burden communities unaccounted for in national and regional control efforts.

To help bridge this knowledge gap, we conducted a rapid survey on soil-transmitted helminth (STH) infections in a small community located in the Honduran Moskitia. Molecular diagnostic techniques based on real-time polymerase chain reaction (qPCR) were employed to provide a more accurate estimate of the prevalence of *N. americanus* and *S. stercoralis*.

## Methods

This study was a non-statistically representative, rapid cross-sectional assessment of STH infections among children and adults in the community of Kaukira, Honduran Moskitia. It provides a snapshot of infection at a single point in time, and all analyses are therefore limited to the enrolled population and not intended to estimate regional prevalence.

### Study site

The department of Gracias a Dios houses approximately 113,000 people (for a population density of 5.9 people per square km). With widespread poverty and limited sources of income, this department ranks number one in all indicators of multidirectional poverty in the country, leading to a subnational human development index of 0.599 [20].

Gracias a Dios can only be reached by air or water, making it difficult for goods and services to be supplied. The difficult access to medical attention for its inhabitants contributes to it having the worst health indicators in Honduras [21–23]. According to the Integrated Food Security Phase Classification (IPC), almost half of the population suffer from serious food insecurity level 3 or higher [19, 22].

**Fig. 1:**
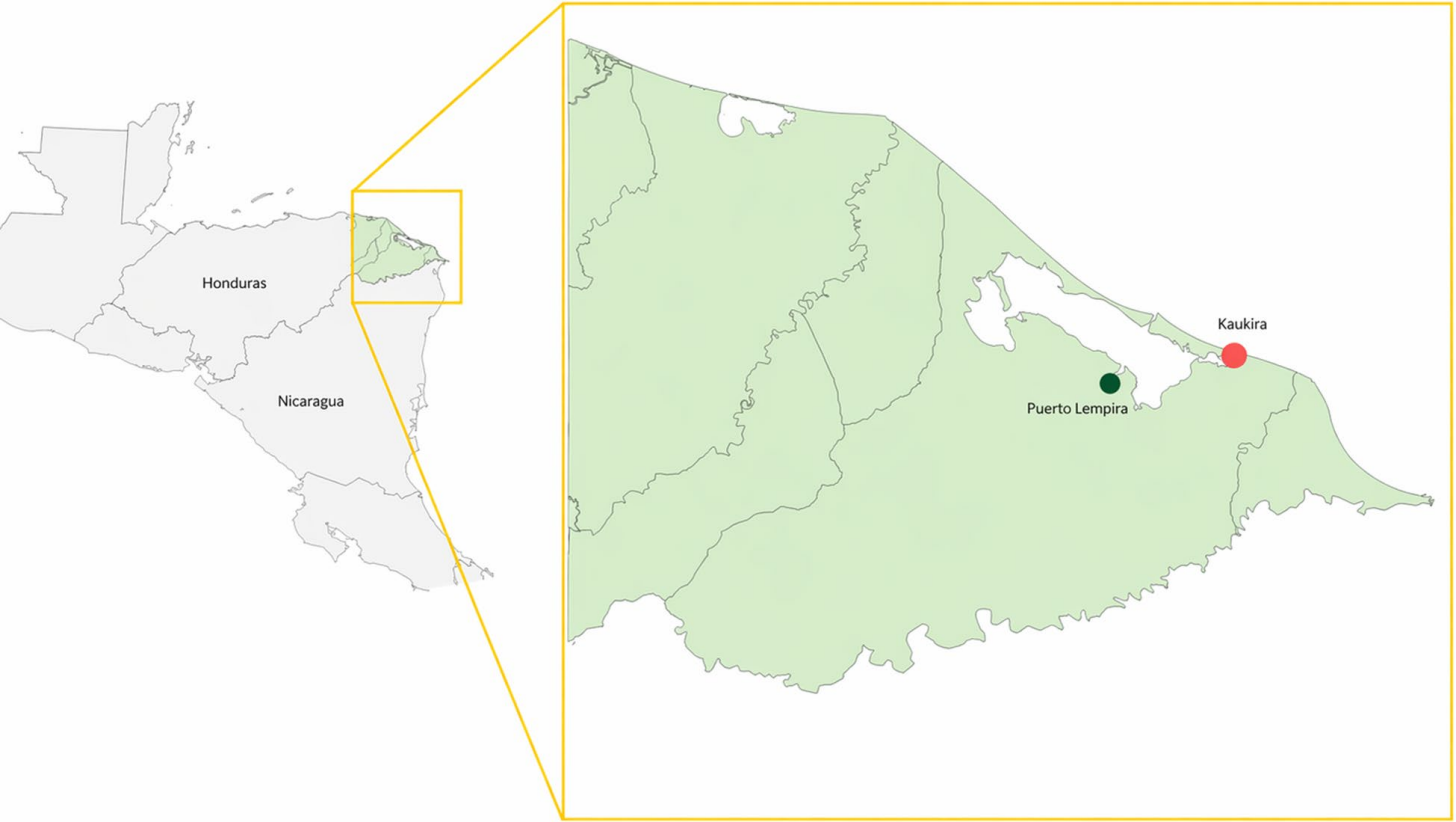
Location of the study site in Kaukira, Gracias a Dios, Honduras. Source: Base map generated using MAGIC Maps 2 for Mac (MAGICMAPS GmbH, Germany). The final figure was produced using Graphic for Mac v3.1 (Picta, Inc.)

Kaukira (also known as Cauquira, 15°19′01.2"N 83°34′58.8"W), is a Miskito village in the municipality of Puerto Lempira, located within the Honduran Moskitia—a vast coastal region spanning both Honduras and Nicaragua [24, 25]. Home to approximately 7000 residents across just over 900 households, Kaukira sits on a narrow strip of land between the Caratasca Lagoon and the Caribbean Sea. Due to its unique geography, access to Kaukira from Puerto Lempira is limited to boat travel across the Caratasca Lagoon (Fig. 1).

### Study participants and samples

To better characterize intestinal parasitism among community members, both school-aged children (SAC, 5–15 years of age) adolescents and adults (≥ 16 years) were invited to participate. Recruitment targeted children enrolled in 1st to 3rd grade at a local primary school as well as adults residing in the surrounding area. Participation was voluntary and each participant was asked to provide a single stool sample. Informed consent or assent were obtained in accordance with age: adults provided written consent; children aged 9 to < 16 years gave oral assent alongside parental consent; and for children under 9 years, both parental consent and child assent were secured.

In the field, two aliquots of 3 g of fecal material from each sample were immediately preserved in 95% ethanol for further molecular analysis. When the amount of sample was sufficient, a third aliquot of 3 g of stools was preserved in 7 ml of 10% buffered formalin. All samples were transported to the Genetic Research Center at the National University of Honduras for analysis.

### The formol-ethyl acetate sedimentation concentration technique

Samples that were preserved in 10% formalin in the field, were processed by the Formol-Ethyl Acetate Sedimentation Concentration Technique (FEAST) [26] and observed under the microscope to identify STH helminth eggs and larvae. Results were recorded qualitatively. Microscopy was only performed in a subset of participants who provided sufficient stool volume and was used as an independent biological confirmation of infection rather than as a reference standard for diagnostic comparison.

### DNA extraction and molecular diagnostics

All samples were processed by qPCR assays. Before processing, samples were washed with distilled water. All samples underwent a pre-extraction treatment to optimize DNA yield. This included one freeze-thaw cycle, where samples were frozen for 30 min at −80 °C and then heated for 10 min at 100 °C. Subsequently, bead-beating was performed for 15 min, using a mix of one stainless steel bead, one rubber bead, and 0.25 mg of 1 μm-diameter glass beads. DNA was extracted using the QIAamp Fast Stool Mini Kit (Qiagen, Hilden, Germany), following the manufacturer’s instructions.

Two multiplex PCR reactions were utilized to detect five STH (*T. trichiura*, *A*. *lumbricoides*, *A. duodenale*, *N. americanus*, and *S. stercoralis*) utilizing primers and probes previously published and validated by Azzopardi and collaborators [27] (Table 1). Reactions were performed using the GoTaq qPCR probe master mix (Promega, USA) in a final volume of 12.5 μl with 2.5 μl of stool DNA. The cycle conditions included a 5-min initial denaturation cycle at 95 °C, followed by 40 cycles at 95 °C for 10 s, and 60 °C for 60 s. The results were visualized with the Mic qPCR Cycler Software (Bio Molecular Systems, Upper Coomera, QLD, Australia).

**Table 1:** Primers and probes used for qPCR assays targeting soil-transmitted helminths

Assay	Parasite	Primer sequence 5´–3´	Target	Reference*
HTS1	*Strongyloides stercoralis*	GAA TTC CAA GTA AAC GTA AGT CAT TAG C	18S rRNA	Modified from [28]
		TGC CTC TGG ATA TTG CTC AGT TC		
		FAM-ACA CAC CGG /ZEN/ CCG TCG CTG C-IBFQ		
	*Trichuris trichiura*	TTG AAA CGA CTT GCT CAT CAA CTT	18S rRNA	Modified from [29]
		CTG ATT CTC CGT TAA CCG TTG TC		
		CY5-CGA TGG TAC /TAO / GCT ACGT GCT TAC CAT GG -IBRQ		
	*Ascaris lumbricoides*	GTA ATA GCA GTC GGC GGT TTC TT	ITS1	Modified from [30]
		GCC CAA CAT GCC ACC TAT TC		
		HEX- TTG GCG GAC /ZEN /AAT TGC ATG CGA T- IBFQ		
HTS2	*Ancylostoma duodenale*	CGG GAA GGT TGG GAG TAT C	ITS1	Modified from [31]
		CGA ACT TCG CAC AGC AAT C		
		HEX-TCG TTAC + T + GGG TGA CGG -IBFQ		
	*Necator americanus*	CTG TTT GTC GAA CGG TAC TTG C	ITS2	Modified from [32]
		ATA ACA GCG TGC ACA TGT TGC		
		CY5- CTG + TA + CTA + CG + CAT + TGT ATA C- IBRQ		

^*^PCR targets were originally described in the referenced source and were implemented in this study following the methodological modifications reported by Azzopardi et al. [27]

Several quality control measures were implemented to ensure the reliability of the qPCR results. Each qPCR run included no-template negative controls and positive controls consisting of stool samples spiked with parasite material, which were subjected to DNA extraction and amplification under the same conditions as study samples. All samples were analyzed in duplicates, in cases of discordant results, a third reaction was performed, and samples were considered positive if amplification was observed in at least two of three reactions. Standard curves generated from serial dilutions of positive control DNA were used to assess assay sensitivity and determine Ct thresholds. Amplification of low-concentration positive controls was detectable between Ct values of 37 and 39, informing the analytical detection limit; however, all positive samples reported in this study had Ct values below 37. Ct values were determined for each sample as the cycle number at which fluorescence exceeded the detection threshold under the same qPCR conditions.

### Statistical analysis

Descriptive statistics were used to summarize participant characteristics. Species-specific and overall prevalence estimates were calculated with 95% confidence intervals (*CI*). Differences in infection proportions between SAC and adults were assessed using Fisher’s exact test. Logistic regression models evaluated associations between infection status, age group, and sex, reporting adjusted odds ratios with 95% *CI*s. For qPCR data, samples with Ct < 40 were considered positive, following established diagnostic thresholds. Among PCR-positive individuals, Ct values were summarized using medians and interquartile ranges and compared between age groups to explore potential differences in relative parasite DNA levels.

Microscopy results were analyzed descriptively and used as an independent parasitological reference to biologically corroborate molecular findings rather than to formally assess diagnostic performance between techniques. Parasite-specific 2 × 2 contingency tables were generated to summarize the agreement between qPCR and microscopy results. In addition, Ct values were compared between microscopy-positive and microscopy-negative samples using non-parametric methods to explore the relationship between molecular signal intensity and microscopic detectability. Given that microscopy data were available only for a subset of participants who provided sufficient stool volume, and that the study was not designed to evaluate diagnostic accuracy, no estimates of sensitivity, specificity, or predictive values were calculated.

## Results

### Overall and specific prevalence

A total of 108 participants (62% female) provided a stool sample, comprising 54 school-age children and 54 adults (Table 2). Among the 108 participants, the qPCR yielded an overall STH prevalence of 85.2% (95% *CI:* 78.62–92.25%) (Table 3). In terms of species prevalence, as shown in Table 3, *T. trichiura* ranked first with 77.8%, followed by *A. lumbricoides* in a distant second place, at 50%. *Necator americanus* ranked third, with a prevalence of 29.6%, while *S. stercoralis* was the least prevalent species, accounting for 3.7%. *A. duodenale* was not detected.

**Table 2:** General characteristics of the study population participating in a parasitological non-representative cross-sectional survey conducted in Kaukira, La Moskitia, Honduras

Characteristic	Overall (*n* = 108*)	SAC (*n* = 54)	Adults (*n* = 54)
Mean age in years (SD^#^)	24.1 (± 20.9)	7.7 (± 1.5)	42.3 (± 16.9)
Sex, female n (%)	67 (62%)	23 (43%)	44 (81%)

^*^15 missing age values were imputed using age group-specific means. ^#^Standard deviation

**Table 3:** Prevalence of PCR-detected STH and median Ct values among PCR-positive individuals participating in a parasitological non-representative cross-sectional survey conducted in Kaukira, La Moskitia, Honduras (*n* = 108)

Parasite	Positive (*n*)	Prevalence (%)	95% confidence interval	Median Ct (IQR)*
*Trichuris trichiura*	84	77.8%	69.1–85.0%	33.9 (32.5–35.8)
*Ascaris lumbricoides*	54	50.0%	40.3–59.7%	29.9 (28.0–31.7)
*Necator americanus*	32	29.6%	21.4–38.9%	33.6 (32.4–35.7)
*Strongyloides* spp.	4	3.7%	1.0–9.1%	31.3 (30.0–33.3)
Overall STH#	92	85.20%	78.62–92.25%	–

^*^Median Ct values are reported among PCR-positive samples only and are presented as a relative indicator of parasite DNA levels. ^#^STH: Soil-transmitted helminth, –: Not applicable.

To characterize relative parasite DNA levels among PCR-positive individuals, Ct values were summarized using medians and interquartile ranges. As shown in Table 4, comparisons were performed using non-parametric methods between Ct obtained from SAC and adults to assess potential differences in Ct distributions between age groups. No statistically significant differences in Ct values were observed between children and adults for any of the detected STH (Wilcoxon rank-sum test; all *P* > 0.05).

**Table 4:** Prevalence of PCR-detected STH by age group with corresponding Ct values among PCR-positive individuals participating in a parasitological non-representative cross-sectional survey conducted in Kaukira, La Moskitia, Honduras (*n* = 108)

	SAC (*n* = 54)		Adults (*n* = 54)		
Parasite	No. of participants (prevalence, 95% *CI*)	SAC median Ct (IQR) ±	No. of participants (prevalence, 95% *CI*)	Adults median Ct (IQR) ±	*P*-value*
Any STH	53 (98.1%, 90.1–100%)	–	39 (72.2%, 58.4–83.5%)	–	< 0.001 **
*Ascaris lumbricoides*	31 (57.4%, 43.2–70.6%)	30.0 (28.3–31.5)	23 (42.6%, 29.3–56.8%)	29.8 (27.2–32.1)	0.178
*Trichuris trichiura*	50 (92.6%, 82.1–98.0%)	34.3 (33.0–36.0)	34 (63.0%, 48.7–75.7%)	33.5 (32.2–35.8)	< 0.001 **
*Necator americanus*	20 (37.0%, 24.3–51.3%)	33.0 (32.3–35.7)	12 (22.2%, 12.1–35.6%)	34.7 (32.8–35.8)	0.140
*Strongyloides stercoralis*	4 (7.4%, 2.1–17.9%)	31.3 (30.0–33.3)	0 (0.0%, 0.0–6.6%)	–	0.118

± Median Ct values with interquartile ranges (IQR) are reported among PCR-positive individuals only. Ct distributions between age groups were compared using the Wilcoxon rank-sum test; no statistically significant differences were observed for any species *P* > 0.05. *P-values refer to prevalence comparisons using Fisher’s exact test. **Statistically significant at *P* < 0.05. SAC: School-age children (5–15 years). Adults: ≥ 16 years, STH: Soil transmitted helminth, –: Not applicable.

### Parasitism, age, and sex

Analysis of the results by age group (Table 4) revealed that except for one child, all were infected with STH (53/54, 98.1%). Furthermore, there was a statistically significant difference in the prevalence of helminth infections between children and adults (Fisher’s exact test, *P* < 0.001). This disparity was primarily attributed to the higher prevalence of *T. trichiura* among children. As shown in Table 4, no significant differences were observed between age groups for the other two species (Fisher’s exact test, *P* > 0.05).

Exploratory logistic regression analyses were conducted to examine associations between infection status and age group (SAC versus adults), adjusting for sex. School-aged children showed higher odds of *T. trichiura* infection compared with adults (adjusted *OR* = 14.55; 95% *CI:* 3.18–66.62; *P* = 0.001), and female sex was also associated with increased odds of infection (adjusted *OR* = 4.36; 95% *CI:* 1.05–18.11; *P* = 0.043). Similarly, SAC showed higher odds of *N. americanus* infection (adjusted *OR* = 3.13; 95% *CI:* 1.18–8.29; *P* = 0.022), while no association with sex was observed (adjusted *OR* = 1.74; 95% *CI:* 0.63–4.83; *P* = 0.289). For *A. lumbricoides*, the association with age group did not reach statistical significance (adjusted *OR* = 2.18; 95% *CI:* 0.90–5.29; *P* = 0.086), and no association with sex was identified (adjusted *OR* = 1.38; 95% *CI:* 0.54–3.54; *P* = 0.504).

### Comparison of diagnostic tests

Microscopy was performed in the subset of participants who provided sufficient stool volume and was used as an independent parasitological method to biologically corroborate molecular findings. Parasite-specific agreement between qPCR and microscopy is summarized in Supplementary Tables S1–S4. For *T. trichiura*, *A. lumbricoides*, and *N. americanus*, microscopy detected infections in a substantial proportion of qPCR-positive individuals, while a smaller number of microscopy-positive/qPCR-negative samples were also observed. *S. stercoralis* was detected exclusively by qPCR, with no microscopy-positive samples identified in this subset.

Among qPCR-positive samples, Ct values were further compared by microscopy status to explore the relationship between molecular signal intensity and microscopic detectability. Median Ct values tended to be lower among microscopy-positive samples, although differences were not statistically significant and substantial overlap in Ct distributions was observed across groups (Supplementary Table S5).

## Discussion

While it is true that there has been a reduction of the global burden due to STH infections [33], these infections are still a public health problem in some regions in Africa and Latin America where they remain negatively correlated with socio-demographic index [6]. Honduras is one of these countries in Central America, where the burden of soil-transmitted helminthiases is still considerable. Similar to other endemic countries, Honduras deploys national deworming campaigns throughout its territory on an annual basis [12]. These efforts can be traced back to the 1990s [2]. Reports from the Honduran ministry of health and the Pan-American Health Organization indicate that Kaukira’s school-aged population receives an annual dose of albendazole 400 mg, and that children between 2–5 years of age receive one dose of mebendazole 500 mg administered during the national immunization week [34].

### Hyperendemicity of soil-transmitted infections

This study identified a high prevalence of STH infections in the community of Kaukira, despite the fact that national mass deworming campaigns have been implemented for more than two decades [35]. Using qPCR, an overall prevalence of 85.2% was observed among enrolled participants, with a high proportion of infections detected among children (98.2%; 95% *CI:* 94.6–100%). While previous studies have consistently reported *T. trichiura* as the most prevalent STH species in Honduras [2, 35, 36], the prevalence levels observed in this study are higher than those previously documented. These findings are consistent with reports from other settings where sustained STH transmission has been observed despite long-standing mass drug administration programs, underscoring the heterogeneity of transmission intensity across endemic communities [37–39].

The high prevalence observed in this study is likely associated with methodological and local factors. Kaukira is an extremely poor and isolated community with inadequate sanitation and waste disposal, and limited access to clean water [25] —conditions that favor persistent transmission [40], as well as the low efficacy of annual single-dose of benzimidazoles as strategy for STH control [35]. In contrast to previous studies restricted to school-aged children, the inclusion of adults in this survey reveals patterns of infection that suggest intergenerational transmission, thus underscoring the need for household-level interventions and age-inclusive surveillance.

Additionally, the use of qPCR, which is markedly more sensitive than microscopy [41], likely increased detection rates.

### *S. stercoralis* and *N. americanus* have been underdiagnosed

The detection of *S. stercoralis* and *N. americanus* highlights the diagnostic advantage of molecular methods. This study found *S. stercoralis* in 3.7% of participants (7.4% among children) and *N. americanus* in 29.6% overall (37% among children)—the highest community-level prevalences reported in Honduras. Previous surveys have shown very low or undetectable levels [12, 42], likely due to the limited sensitivity of microscopy-based Kato-Kats method. Similarly, past field studies reported hookworm prevalences of 2.0–5.1% [2, 35], with the highest report of 15.9% when slides were promptly read [11]. Comparable *S. stercoralis* rates in Honduras have only been observed in a clinical study in HIV-positive participants [43, 44].

In Honduras, diagnostic limitations and the exclusion of adults have likely led to the underrepresentation of these parasites in STH epidemiological research [41, 45]. As infection intensity declines due to regular mass-deworming, the limitations of Kato-Katz sensitivity become evident. Hence, integrating molecular DNA-based diagnostics is essential. These methods yield more accurate prevalence estimates and strengthen monitoring of deworming efficacy, enabling data-driven and sustainable STH control strategies [46, 47].

### Microscopy findings as complementary parasitological evidence

In this study, Kato-Katz examination could not be implemented under recommended conditions because stool samples were not processed in the field, reflecting logistical constraints associated with the remoteness of the study area. Instead, a fecal concentration technique was used to identify parasite diagnostic stages and to provide independent parasitological corroboration of molecular findings. Microscopy confirmed the presence of helminth infections in a subset of participants and supported the existence of active transmission within the study community. Variability in agreement between microscopy and qPCR was observed, consistent with the different analytical characteristics of the two approaches [48].

### Demographic factors and infection intensities

Exploratory analyses suggested age- and sex-related patterns for certain STH species within the enrolled population. School-aged children exhibited higher odds of infection with *T. trichiura* and *N. americanus*, whereas no statistically significant association was observed for *A. lumbricoides*. Female sex was associated with *T. trichiura* infection in this dataset. These observations are broadly consistent with previous studies that have identified children as a group frequently affected by STH infections [39, 49]. However, given the limited sample size, voluntary participation, and recruitment of children from a single school, these associations should be interpreted with caution and are best viewed as exploratory and hypothesis-generating rather than as evidence of population-level risk patterns. More systematic, population-based studies will be required to robustly assess age- and sex-related differences in STH distribution.

Analyses of Ct values among PCR-positive individuals did not identify statistically significant differences between children and adults for any of the detected species, indicating largely comparable Ct distributions across age groups within the study population. Although adults were slightly less frequently infected, the similarity in Ct values suggests that, among infected individuals, relative parasite DNA levels were comparable between age groups. These findings underscore the importance of extending parasitological surveys to include adults as well as children, as the older members of the community could act as previously overlooked parasite reservoirs. They also highlight the need for adequately powered longitudinal studies to better evaluate age-related patterns in infection dynamics [40].

Limitations should be considered when interpreting these results. The relatively small sample size and voluntary participation limit the generalizability of the findings, and interpretations should therefore be restricted to the enrolled population. In addition, while the qPCR assay applied has been previously validated and multiple internal quality control measures were implemented, the absence of an internal amplification control and participation in an external inter-laboratory quality assessment scheme limits formal evaluation of extraction efficiency, inhibition, and inter-laboratory comparability. Together, these limitations highlight the need for optimized and harmonized molecular protocols for STH assessment. Nonetheless, the data provides evidence of saturated transmission dynamic within the study community where STH infection is near-universal for the pediatric population. The results also underscore the importance of conducting larger, statistically powered parasitological surveys and expanded molecular investigations to more comprehensively characterize STH epidemiology in the region.

## Conclusions

This study provides valuable information on the presence and distribution of STH infections within the studied community, contributing local data from a setting where epidemiological information remains limited. The findings indicate not only ongoing transmission among the enrolled population with near universal infection in children and highlight the value of incorporating molecular approaches to determine infection patterns in highly endemic contexts.

While the results should be interpreted within the limitations of the study, they are consistent with long-standing observations that environmental and social conditions play an important role in sustaining STH transmission. In this sense, the data from Kaukira support the need for continued parasitological surveillance and for larger, more comprehensive studies to better understand transmission dynamics and inform future control efforts in endemic regions.

## Supplementary Information

Supplementary Material 1.

## Data Availability

All datasets generated and analyzed during this study are included within the manuscript. No additional data are available.

## Abbreviations

STH: Soil-transmitted helminths
FEAST: Formalin-ethyl acetate sedimentation technique
KK: Kato-Katz
MDA: Mass drug administration
PCR: Polymerase chain reaction
qPCR: Real-time PCR
WHO: World Health Organization
DNA: Deoxyribonucleic acid
