# Supplementary material for: Heavy burden of soil-transmitted helminth infections in a remote and impoverished indigenous community of Honduras revealed by real time polymerase chain reaction
Source: Infect Dis Poverty. 2026 May 25;15:61. doi: 10.1186/s40249-026-01454-5 (PMC13200298; doi:10.1186/s40249-026-01454-5)
Supplement: Supplementary file 1 — Supplementary Material 1. [file 40249_2026_1454_MOESM1_ESM.docx]

Supplementary material

Supplementary Table 1. Contingency table summarizing agreement between qPCR and microscopy for *Necator americanus.*

|  | Microscopy positive | Microscopy negative | Total |
| --- | --- | --- | --- |
| qPCR positive | 5 | 25 | 30 |
| qPCR negative | 0 | 73 | 73 |
| Total | 5 | 98 | 103 |

Supplementary Table 2. Contingency table summarizing agreement between qPCR and microscopy for *Ascaris lumbricoides.*

|  | Microscopy positive | Microscopy negative | Total |
| --- | --- | --- | --- |
| qPCR positive | 39 | 12 | 51 |
| qPCR negative | 5 | 47 | 52 |
| Total | 44 | 59 | 103 |

Supplementary Table 3. Contingency table summarizing agreement between qPCR and microscopy for *Trichuris trichiura.*

|  | Microscopy positive | Microscopy negative | Total |
| --- | --- | --- | --- |
| qPCR positive | 55 | 25 | 80 |
| qPCR negative | 2 | 21 | 23 |
| Total | 57 | 46 | 103 |

Supplementary Table 4. Contingency table summarizing agreement between qPCR and microscopy for *Strongyloides stercoralis.*

|  | Microscopy positive | Microscopy negative | Total |
| --- | --- | --- | --- |
| qPCR positive | 0 | 4 | 4 |
| qPCR negative | 0 | 99 | 99 |
| Total | 0 | 103 | 103 |

| Parasite | Microscopy status (qPCR-positive only) | n | Median Ct (IQR) | p-value* |
| --- | --- | --- | --- | --- |
| *N. americanus* | positive | 5 | 32.4 (30.1-34.0) | 0.121 |
|  | negative | 25 | 33.3 (32.5-35.7) |  |
| *A. lumbricoides* | positive | 39 | 29.9 (27.7-31.1) | 0.074 |
|  | negative | 12 | 32.2 (28.5-33.5) |  |
| *T. trichiura* | positive | 55 | 33.8 (32.3-35.4) | 0.368 |
|  | negative | 25 | 33.9 (32.5-36.0) |  |
| *S. stercoralis* | positive | 0 | - | - |
|  | negative | 4 | 31.3 (30.0-33.3) |  |

Supplementary Table S5. Ct values by microscopy status among qPCR-positive samples

*p-values were calculated using the Wilcoxon rank-sum test (exact) to compare Ct distributions between microscopy-positive and microscopy-negative samples. -For *Strongyloides stercoralis*, comparisons were not performed due to the absence of microscopy-positive samples.
